# Sex Differences in the Association between Living Environmental Factors and Nutritional Status in Community-Dwelling Elderly Koreans

**DOI:** 10.3390/ijerph17176034

**Published:** 2020-08-19

**Authors:** Dong Eun Kim, Hee-Sook Lim, Hyejin Ahn, Young Sun Kim, Yoo Kyoung Park

**Affiliations:** 1 Department of Medical Nutrition, Graduated School of East-West Medical Science, Kyung Hee University, Yongin 17104, Korea; kim-doeu@hanmail.net (D.E.K.); hjahn@khu.ac.kr (H.A.); 2 Department of Food Sciences and Nutrition, Yeonsung University, Anyang 14011, Korea; limhs@yeonsung.ac.kr; 3 New Aging Center, Kyung Hee University, Yongin 17104, Korea; 4 Department of Gerontology, Graduate School of East-West Medical Science, Kyung Hee University, Yongin 17104, Korea

**Keywords:** elderly, nutrition quotient for elderly (NQ-E), dietary behaviors, living environment, social activity

## Abstract

The association between nutritional status and living environment among 703 community-dwelling participants (268 men and 435 women) aged 65 years and older was assessed. In this cross-sectional survey study, living environmental factors, health-related factors, and nutrition (the Nutrition Quotient for the Elderly scores; NQ-E) were assessed. NQ-E scores were significantly higher in men than women, as were diversity and behavior factor scores (diversity: men, 50.2 ± 16.1; women, 44.1 ± 17.5; behavior: men, 59.3 ± 16.9; women, 54.1 ± 16.6). Participants living with a spouse and engaging in frequent sports activities had significantly higher adjusted odds ratio (OR) for having a high NQ-E than those who lived alone and engaged in restful activities and hobbies (men: adjusted OR for high NQ-E = 8.99; 95% confidence interval (CI): 1.35–59.56; women: adjusted OR for high NQ-E = 5.62; 95%CI: 2.36–13.38). We confirmed that women’s nutritional status, unlike men’s, was better when proper nutrition education was provided and when food security was guaranteed. For all participants, social activities and networks were important for maintaining good nutritional status and a healthy life. We suggest that different nutritional management strategies are needed for elderly people depending on sex.

## 1. Introduction

A super-aged society, where more than 20% of the total population is aged 65 years and older, is in our near future and represents an emerging global challenge [1]. Korea is expected to have the highest percentage of elderly people in the world, with the population composition of those aged 65 or older calculated to reach 43.9% in 2060, up from 14.9% in 2019 [2]. The European Union is emphasizing promotion of a healthy “active aging” society and active lifestyles among older adults [3]. As elderly peoples’ activities and social participation increase, the desire to pursue values and to improve quality of life rather than to simply hope for survival is growing [4]. Aging is defined as the collective series of physiological changes that occur in an organism over time, resulting in progressive deterioration of functioning, increased vulnerability to disease, and reduced viability [5,6]. Specifically, various factors such as poor appetite, restricted activity, reduced income, social isolation, and depression cause many changes in physiological functioning and health conditions [7]. In addition, medication, hospitalization, and social aspects of aging can also contribute to nutritional inadequacy [8]. A cornerstone for healthy aging is optimal nutritional status. Early identification of older adults who are at risk for insufficient caloric intake and nutrient adequacy, termed nutritional risk, is paramount to maintaining health, independence, quality of life, and longevity [9,10]. Most studies have reported sex-specific differences in the nutritional status and community integration of elderly individuals. Kiefer et al. [11] suggested that women have greater awareness and better knowledge of nutrition and nutrient intake than men. By contrast, women are reported to have more eating problems and greater difficulty managing diets [12]. These differences indicate that numerous factors such as education, marital status, household composition, socioeconomic status, social support, and geographic and environmental characteristics affect nutritional status and eating behaviors [13,14]. Korea’s poverty ratio of the old is higher than that of Organization for Economic Cooperation and Development (OECD) countries, and Korean elderly women have lower education and economic level than men and are more likely to live alone [15,16]. Women have a higher rate of sarcopenia and lower nutritional status than men due to the higher proportion of women who lack exercise [17]. Food diversity in sarcopenic women was also reported to be lower than in the non-sarcopenia population [15]. This suggests that nutritional status and dietary behaviors can be influenced by psychological and sociocultural factors and that the effects may vary by sex. Therefore, there is a need for better understanding the aspects of older men and women’s living environments that affect nutritional status, dietary behavior, and dietary practice.

This study examined the differences between health-related conditions and environmental factors according to sex in community-dwelling elderly Koreans and the social environmental factors affecting nutritional status.

## 2. Materials and Methods

### 2.1. Study Subjects

A cross-sectional survey study was conducted in elderly community-dwelling Koreans between July 2019 and December 2019. The inclusion criteria were as follows: Korean nationality, current residence in Korea, and ≥65 years old with no visual disturbance or severe vision impairment. Those who were illiterate, unable to understand texts, or unable to communicate were excluded. Simple random sampling and regional sampling were used as a sampling technique considering the regional distribution of subjects. We determined the sample size to be 700 people, based on a 99% confidence interval with a margin of error of 5% in 850 million persons aged 65 years or over in Korea in 2019 [18,19]. Using a self-administered questionnaire, 794 questionnaires were collected, and 703 questionnaires were ultimately used; 91 were excluded due to missing information or refusal to participate. This study was approved by the Institutional Review Board of Kyung Hee University under the Helsinki Research Principles (approval number: KHSIRB-19-005).

### 2.2. Assessing Nutritional Status

Participants’ nutritional status was assessed using the Nutrition Quotient for Elderly (NQ-E), an evaluation tool for measuring food behavior and dietary quality in people aged 65 years or older. The NQ-E was developed and validated by the Korean Nutrition Society in 2018 based on the literature and on data from the Korean National Health and Nutrition Examination Survey [20]. The NQ-E questionnaire consists of 19 items categorized into four factors: “dietary behavior” (6 items addressing difficulties in chewing foods, perception of one’s health, depressive symptoms, handwashing practices before eating meals, hours of exercise, and efforts to have healthy eating habits); “balance” (4 items addressing intake frequencies of milk or dairy products, fruits, snacks, and water); “diversity” (6 items covering intake frequencies of eggs, fishes or shellfishes, and beans or bean products; frequency of eating alone; number of vegetable dishes at each meal, excluding kimchi; and meal frequency); and “moderation” (3 items covering intake frequency of sweets and baked products, sugar-added beverages, and instant noodles). The weighted scores for all four factors were added, yielding the total NQ score, which ranged from 0 to 100. The participants were divided into 3 groups categorized by the Korean Nutritional Society [20] according to the total NQ score (high: 63.5–100, medium: 51.9–63.4, and low: 0–51.8).

### 2.3. Demographic Characteristics, Body Mass Index (BMI), and Health-Related Factors

Demographic characteristics such as age, sex, employment status, education, and monthly income were recorded. Body mass index (BMI) was calculated based on height and weight. This study used weight categories from the World Health Organization (WHO) Asia-Pacific Perspective and the Korean Society for the Study of Obesity: underweight and normal, ≤22.99 kg/m^2^; overweight, 23.0–24.99 kg/m^2^; and obese and severely obese, ≥25.00 kg/m^2^ [21]. The number of chronic diseases (hypertension, dyslipidemia, stroke, myocardial infarction, arthritis, osteoporosis, diabetes mellitus, cancers, and digestive disorders) and medications, activities of daily living (ADL), usual form of transportation (walking only, bus, subway, car or taxi, and so forth), social support (social care services, home delivery meal service, and free meal service), and nutrition counseling experiences were recorded as health-related factors. 

### 2.4. Living Environmental Factors Related to Food Intake

Living environmental factors included social and food intake-related factors. Living status (alone, with spouse, and with children or children’s families), social activities (resting and hobbies, cultural and artistic, sports, or other activities), frequency of social activity (rarely, regular, and trying to be active), and social relations (at least 3 times per week, 1–2 times per week, fewer than 3 times per month, and almost none) were investigated as social factors. Food security (secure, mildly secure, and moderately or severely insecure), ability to cook, and nutrition knowledge (low, medium, and high) were assessed as food intake-related factors. Food security was evaluated using a three-item measure of food insecurity during the last 1 year: (1) “Do you have enough money to buy food you need most of the time?”; (2) “In the past year, have you skipped one or more meals because you had no food in the house or because you thought that you might not have enough food soon?”; and (3) “In the past year, have you had to choose between buying food and paying bills or buying something else you needed?” [22]. The nutrition knowledge questionnaire consisted of 10 true–false questions referring to prior research [23]: proteins have the most caloric nutrients, vitamins are calorie nutrients, fats are a nutrient that strengthens bones, soybeans have a lot of protein, milk is the best source of calcium, iron deficiency causes anemia, egg yolks contain a lot of cholesterol, sufficient water intake prevents constipation, people with hypertension should avoid salty foods, and fiber is a caloric nutrient. Scores were considered to indicate high (8–10 points), medium (4–7 points), and low (0–3 points) nutrition knowledge depending on the number of correct answers.

### 2.5. Statistical Analyses

Subjects’ demographic characteristics and dietary factors are presented as mean and standard deviation for continuous variables and as frequency and percentage (%) for categorical variables. Before conducting comparative analysis between groups, we identified that the data had a normal distribution. Group comparisons used chi-square tests for qualitative variables and independent *t*-tests or one-way analysis of variance (ANOVA) for quantitative variables. Subsequently, multinomial logistic regression analyses were performed to evaluate factors affecting the NQ-E. All analyses were carried out using the Statistical Package for Social Sciences for Windows version 25.0 (SPSS, Inc., Chicago, IL, USA). Statistical significance was set at 0.05 based on a two-sided test.

## 3. Results

### 3.1. Demographic Characteristics

Participants’ demographic characteristics are summarized in Table 1. The mean age was 74.4 ± 6.4 years (men, 74.3 ± 6.2; women, 74.4 ± 6.6 years). The level of education most frequently achieved was college or higher for men (36.9%) and elementary school graduation (35.6%) for women (*p* < 0.001). The most common employment status for both men and women was unemployed (men, 79.9%; women, 87.4%; *p* < 0.01), and the most frequent income level for both was <500,000 won (men, 24.3%; women, 39.8%; *p* < 0.001).

### 3.2. Comparision of NQ-E Total and Component Scoress by Sex

Subjects’ NQ-E scores are shown in Table 2. The mean NQ-E score was 58.7 ± 11.1. The mean NQ-E was significantly higher for men than for women (59.9 ± 10.6 vs. 58.1 ± 11.4, respectively; *p* < 0.05). Men’s scores on the diversity and behavior factors of the NQ-E were significantly higher than women’s (diversity: 50.2 ± 16.1 vs. 44.1 ± 17.5, respectively; *p* < 0.001; behavior: 59.3 ± 16.9 vs. 54.1 ± 16.6, respectively; *p* < 0.001). The balance and moderation factors did not differ by sex. When compared by age, as the age of men and women increase, the average NQ score was decreased. Men showed significant differences in NQ-E, diversity, and behavior, while women showed differences in NQ-E, balance, diversity, moderation, and behavior.

### 3.3. Sex Differences in Health-Related and Living Environmental Factors According to NQ-E Level

Sex differences in health-related and living environmental factors according to NQ-E are presented in Table 3 and Table 4. In both men and women, significant differences were found according to NQ-E level (high, medium, and low) in the number of diseases and medications, ADL, current living status, social relations and activities, transportation, experience of social support by government, food security, and nutrition knowledge.

Participants with low NQ-E had the highest number of diseases and medications (men, *p* = 0.003; women, *p* = 0.001). Men and women with high NQ-E scores had the highest rate of living with their spouse (men, 91 (91.0%); women, 91 (65.5%)). Women with low NQ-E had the highest rate of living alone (89 (65.9%)). Men and women with high NQ-E had the highest number of social relations (at least 3 times/week), engagement in sports activities, and frequency of social activities. In terms of food security among both men and women, those with high NQ-E scores had the highest food security and nutrition knowledge. Women with high NQ-E had the highest cooking ability and the most nutritional counseling experience. For men, these factors did not vary significantly according to NQ-E scores. 

### 3.4. Effects of Living Environment on NQ-E by Sex: Multinomial Logistic Regressions

The effects of living environments on NQ-E levels according sex are shown in Table 5 and Table 6. Men and women living with a spouse had a significantly higher adjusted odds ratio (OR) for high NQ-E levels than those who were living alone (men: adjusted OR for high NQ-E = 8.99; 95% confidence interval (CI): 1.35–59.56; women: adjusted OR for high NQ-E = 5.62; 95%CI: 2.36–13.38). Elderly men and women who participated in sports activities had a significantly higher adjusted OR for medium or high NQ-E levels compared to those who engaged in sedentary and hobby activities (men: adjusted OR for medium NQ-E = 3.41; 95%CI: 1.18–9.80; women: adjusted OR for high NQ-E = 12.07; 95%CI: 3.91–37.20). Among women, those who had social interactions at least 3 times/week had a significantly higher adjusted OR of high NQ-E levels than those with fewer social interactions (adjusted OR for high NQ-E = 16.04; 95%CI: 1.72–149.18). 

## 4. Discussion

This study examined the association between NQ-E and various elements of one’s living environment including health-related factors, food- and nutrition-related factors, social activity, and mobility factors by sex. Social activity and networks were important to good nutritional status and a healthy life in all participants. The results of NQ-E were closely related to one’s living environment. NQ-E in both men and women was particularly closely associated with living status and social activities. Furthermore, the nutrition quotient of elderly women was additionally related to various living environmental factors such as social relations, food security, and nutrition knowledge. We suggest that it may be helpful to consider sex differences in dietary management for elderly people. 

Based on the NQ-E results among the male participants, high scores on dietary behavior and diversity are thought to be related to social support. Social support of various types is associated with positive health outcomes [24]. Importantly, whether one is living alone or not, the main factor for evaluating social support in elderly people is malnutrition [7,25]. Not surprisingly, living with a spouse was an important factor in social and emotional stability. Older adults’ closest emotional connections are with their spouses or partners [7,14]. Moreover, life satisfaction is also high among elderly people who live a spouse-oriented life [7,14,25]. Older people living with their spouses usually have well-managed health care and better nutritional status than those who live alone [26,27,28]. In this study, the rate of living with a spouse was twice as high in men as in women. It is thought that this difference played a role in men’s diverse food intake and high NQ-E score. This result suggests that older people who live alone may need social care services that provide social and emotional support, both for nutrition management and good nutritional status and for overall health.

In this study, women’s low NQ-E scores were primarily due to low scores for dietary behavior, which includes questions about difficulties in chewing foods, perception of one’s health, depressive symptoms, handwashing practices before eating meals, hours of exercise, and efforts to have healthy eating habits. Among the participants in this study, 40.9% of elderly women was living alone, which was higher than the 27.4% reported in another study [29,30]. Thus, it may be that women reported more depressive feelings because of the length of time they lived alone, which tends to be longer for women than for men due to differences in life expectancy. In addition, women’s job levels and income are often lower than those of men, which may contribute to depression among elderly women. Therefore, it is thought that these components of dietary behavior among the women in this study may partially explain the lower NQ-E scores. Because exercise reduces depression, sports activities are often recommended to relieve its effects [2,31]. However, elderly women are also known to have more difficulty participating in exercise than men. 

In both elderly men and women, NQ-E was positively associated with living with one’s spouse and with engaging in a fair amount of social activity. In women, however, social relations, food security, and nutrition knowledge were additionally related to NQ-E, suggesting that various elements of the living environment are more closely linked to nutrition in women than in men. For these reasons, we need to focus on increasing older people’s social networks. Recently, the importance of social networks for a healthy life has emerged in addition to physical health [24,31]. However, in reality, elderly people tend to have a limited social network after a spouse’s death, which may be compounded by economic problems and retirement from work [7,14]. Social networks have also been reported to affect depression in elderly people, a major factor influencing food intake [7]. Therefore, social networks are very important for improving nutritional status among elderly people as well as their social health and quality of life. Kim et al.’s findings support the conclusion that the better the social network is, the lower the nutritional risk will be [14,24].

In the present study, elderly men living with a spouse had higher nutrition quotients than did women with the same living status. This is likely due to the large influence of a spouse on men’s social networks. Men who live alone reported having poorer meal quality and lower food intake than men who lived with a spouse [32]. In addition, men who lived alone had high intake of convenience foods and high-fat/high-cholesterol meals [33]. This means that living with a spouse is a very important factor in food intake and nutritional status. 

Women with higher NQ-Es had the highest rates of living with a spouse and of engaging in social activities. In other words, women with more frequent social relations and activities and with greater food security and nutritional knowledge had higher NQ-E scores than women with lower values in those areas. Thus, elements of the living environment were more closely linked to women’s NQ-E levels than to that of men. Therefore, a multicomponent approach is needed to improve the nutritional status and health of elderly women. These results suggest that the social health and nutritional status of both men and women will be improved by encouraging social activities, such as sports activities, that can create a social network. In addition, a combined approach that addresses multiple factors, such as nutrition education and social activities, may be needed to improve elderly women’s nutritional status and health. 

When women’s nutritional knowledge score was medium or high, their nutritional status was significantly better, suggesting that being health conscious may improve nutritional status. Women were most interested in the topics of health and happiness and they participated in more educational programs, social services, and cultural activities than did men [34]. Concern about health and nutritional knowledge in women reportedly influences desirable eating habits [35]. Women’s high level of interest in health may be related to their chronic diseases. This suggestion is in line with Timpini et al. [36], who found that women had a higher prevalence of disease than men. In the present study, the average number of diseases reported by elderly women with low NQ-E scores was higher than that in elderly men with similar NQ-E scores. Thus, continuous nutrition education and management support for women are likely to greatly improve their health. These findings suggest that chronic disease-related nutrition education will be very useful in enhancing dietary behavior and nutritional status. 

Some limitations of this study should be noted. First, the cross-sectional nature of the study limits our ability to invoke causal relationships between the nutrition quotient and elements of the participants’ living environments. Second, since the study was conducted as a self-administered questionnaire, we had to exclude the elderly with poor literacy. This may have excluded some older people who may be more nutritionally vulnerable. Third, the uncertainty level is high for some factors, as indicated by the wide confidence intervals seen in the results of the multinomial logistic regression analyses. This seems to have resulted from the low numbers of participants in some categories; hence, longitudinal studies of the relevant factors are necessary. Meanwhile, the main strength of this study is that the results were obtained using data collected through large-scale nationwide surveys of elderly people in various regions of the country. It is also meaningful that the study analyzed various factors of the living environment according to sex, which can clearly affect nutritional status in elderly community-dwelling people.

## 5. Conclusions

We examined the associations between food quality, food behavior, and nutritional status and living environmental factors in Koreans over 65 years old using the NQ-E. NQ-E scores, including dietary behavior and diversity, of elderly men were significantly higher than those of elderly women. The living situations and social activities of both men and women were related to nutritional status. In particular, elderly individuals living with a spouse and those who played sports had higher nutrition quotients than those who lived alone and those with sedentary lives. Meanwhile, women who experienced food security and had high levels of nutrition knowledge showed higher nutrition quotients than those experiencing moderate or severe food insecurity and low nutrition knowledge. Our results confirm the existence of sex differences in the relationship between the nutrition quotient and living environmental factors such as social activity and support systems. It is important to educate elderly individuals about the role of social networks and relationships in improving their nutritional status. Elderly people should be provided with opportunities for social activities to improve their nutritional status.

## Figures and Tables

**Table 1:** General characteristics of the subjects by sex.

	Total (*n* = 703)	Sex								
		Men (*n* = 268)				Women (*n* = 435)				*p*-Value ^(3)^
Average age, mean ± SD	74.38 ± 6.41	74.32 ± 6.17				74.41 ± 6.55				0.869
**Age**		**65–74** **(*n* = 148)**	**75–84** **(*n* = 101)**	**≥85** **(*n* = 19)**	***p*-Value ^(2)^**	**65–74** **(*n* = 233)**	**75–84** **(*n* = 164)**	**≥85** **(*n* = 38)**	***p*-Value ^(2)^**	***p*-Value ^(4)^**
Education, *n* (%)										
Uneducated	94 (13.4)	5 (3.4)	7 (6.9)	4 (21.1)	0.000	18 (7.7)	45 (27.4)	15 (39.5)	0.000	0.000
Elementary school	198 (28.2)	12 (8.1)	25 (24.8)	6 (31.6)		79 (33.9)	58 (35.4)	18 (47.4)		
Middle school	97 (13.8)	14 (9.5)	8 (7.9)	2 (10.5)		53 (22.7)	20 (12.2)	0 (0.0)		
High school	173 (24.6)	58 (39.2)	25 (24.8)	3 (15.8)		51 (21.9)	32 (19.5)	4 (10.5)		
College or Higher	141 (20.1)	59 (39.9)	36 (35.6)	4 (21.1)		32 (13.7)	9 (5.5)	1 (2.6)		
Employment, *n* (%)										
Employed	109 (15.5)	41 (27.7)	12 (11.9)	1 (5.3)	0.002	40 (17.2)	12 (7.3)	3 (7.9)	0.010	0.008
Unemployed	594 (84.5)	107 (72.3)	89 (88.1)	18 (94.7)		193 (82.8)	152 (92.7)	35 (92.1)		
Monthly income, *n* (%) ^(1)^										
<50 50–99 100–199 200–299 ≥300	238 (33.9) 179 (25.5) 111 (15.8) 77 (11.0) 98 (13.9)	19 (12.8) 26 (17.6) 38 (25.7) 18 (12.2) 47 (31.8)	34 (33.7) 22 (21.8) 19 (18.8) 13 (12.9) 13 (12.9)	12 (63.2) 4 (21.1) 2 (10.5) 1 (5.3) 0 (0.0)	0.000	59 (25.3) 74 (31.8) 36 (15.5) 36 (15.5) 28 (12.0)	87 (53.0) 44 (26.8) 15 (9.1) 8 (4.9) 10 (6.1)	27 (71.1) 9 (23.7) 1 (2.6) 1 (2.6) 0 (0.0)	0.000	0.000

SD = standard deviation; ^(1)^ units = 10,000 won; ^(2)^ *p*-values were expressed between age using a chi-square test.; ^(3)^ *p*-values were expressed between sex using an independent *t*-test; and ^(4)^ *p*-values were expressed between sex a chi-square test or independent *t*-test.

**Table 2:** The level and scores of the Nutrition Quotient for Elderly (NQ-E) by sex.

		Total	Sex								
			Men (*n* = 268)				Women (*n* = 435)				
Age			65–74 (*n* = 148)	75–84 (*n* = 101)	≥85 (*n* = 19)	*p*-Value ^(3)^	65–74 (*n* = 233)	75–84 (*n* = 164)	≥85 (*n* = 38)	*p*-Value ^(3)^	*p*-Value ^(4)^
**NQ-E**	Low ^(1)^	195 (27.7)	62 (41.9)	36 (35.6)	2 (10.5)	0.000	89 (38.2)	45 (27.4)	5 (13.2)	0.000	0.042
	Medium	269 (38.3)	66 (44.6)	35 (34.7)	7 (36.8)		98 (42.1)	52 (31.7)	11 (29.0)		
	High	239 (34.0)	20 (13.5)	30 (29.7)	10 (52.6)		46 (19.7)	67 (40.9)	22 (57.9)		
Average NQ-E score ^(2)^, mean ± SD		58.74 ± 11.12	61.98 ± 9.77	58.11 ± 11.33	52.65 ± 8.57	0.000	60.60 ± 10.31	55.90 ± 12.29	51.85 ± 9.25	0.000	0.037
**Balance**	Low ^(1)^	234 (33.3)	41 (27.7)	29 (28.7)	2 (10.5)	0.428	77 (33.0)	40 (24.4)	5 (13.2)	0.000	0.425
	Medium	275 (39.1)	59 (39.8)	32 (31.7)	8 (42.1)		109 (46.7)	51 (31.1)	16 (42.1)		
	High	194 (27.6)	48 (32.4)	40 (39.6)	9 (47.4)		47 (20.2)	73 (44.5)	17 (44.9)		
Average balance score, mean ± SD		39.29 ± 23.27	40.67 ± 21.45	36.88 ± 23.63	38.40 ± 22.34	0.062	44.33 ± 22.61	35.54 ± 25.08	30.93 ± 19.88	0.000	0.424
**Diversity**	Low	249 (35.4)	36 (24.3)	37 (36.6)	3 (15.8)	0.001	67 (28.8)	24 (14.6)	3 (7.9)	0.000	0.000
	Medium	284 (40.4)	87 (58.8)	35 (34.7)	6 (31.6)		101 (43.4)	48 (29.3)	2 (5.3)		
	High	170 (24.2)	25 (16.9)	29 (28.7)	10 (52.6)		65 (27.9)	92 (56.1)	28 (73.7)		
Average diversity score, mean ± SD		46.45 ± 17.20	51.82 ± 12.84	50.11 ± 18.91	38.66 ± 18.13	0.003	48.81 ± 16.37	39.85 ± 17.71	33.87 ± 14.20	0.000	0.000
**Moderation**	Low	114 (16.2)	55 (37.2)	45 (44.6)	9 (47.4)	0.119	104 (44.6)	95 (57.9)	23 (60.5)	0.055	0.028
	Medium	258 (36.7)	70 (47.3)	32 (31.7)	8 (42.1)		89 (38.2)	48 (29.3)	11 (19.0)		
	High	331 (47.1)	23 (15.5)	24 (23.8)	2 (10.5)		40 (17.2)	21 (12.8)	4 (10.5)		
Average moderation score, mean ± SD		82.55 ± 16.06	81.97 ± 14.77	79.24 ± 19.44	84.43 ± 17.19	0.308	81.63 ± 16.16	85.60 ± 14.45	85.18 ± 15.01	0.033	0.063
**Behavior**	Low	196 (27.9)	77 (52.0)	37 (36.6)	2 (10.5)	0.000	86 (36.9)	33 (20.1)	5 (13.2)	0.000	0.000
	Medium	267 (38.0)	50 (33.7)	33 (32.7)	7 (36.9)		96 (41.2)	71 (43.3)	10 (16.3)		
	High	240 (34.1)	21 (14.2)	31 (30.7)	10 (52.6)		51 (21.9)	60 (36.6)	23 (60.5)		
Average behavior score, mean ± SD		56.09 ± 16.89	62.97 ± 15.43	56.49 ± 17.50	46.15 ± 15.75	0.000	58.23 ± 15.56	50.46 ± 16.32	44.47 ± 16.73	0.000	0.000

NQ-E = nutritional quotient for elderly; ^(1)^ data are expressed as frequencies and percentages (%); ^(2)^ data are express as means± standard deviations (SD); ^(3)^ *p*-values were expressed between age using a chi-square test or independent *t*-test; and ^(4)^ *p*-values were expressed between sex a chi-square test or independent *t*-test.

**Table 3:** Health-related factors of the subjects according to NQ-E level.

	NQ-E levels (*n* = 703)							
	Men (*n* = 268)				Women (*n* = 435)			
	Low (*n* = 60)	Medium (*n* = 108)	High (*n* = 100)	*p*-Value	Low (*n* = 135)	Medium (*n* = 161)	High (*n* = 139)	*p*-Value
BMI ^(1)^, *n* (%)								
≤Normal	32 (53.3)	35 (32.4)	25 (25.0)	0.007	67 (49.6)	62 (38.5)	64 (46.0)	0.283
Overweight	14 (23.3)	42 (38.9)	42 (42.0)		36 (26.7)	46 (28.6)	33 (23.7)	
≥Obesity	14 (23.3)	31 (28.7)	33 (33.0)		32 (23.7)	53 (32.9)	42 (30.2)	
Average BMI, mean ± SD	22.98 ± 2.83	23.75 ± 2.44	24.07 ± 2.34	0.030	22.97 ± 3.20	24.00 ± 3.20	23.78 ± 3.00	0.015
Numbers of diseases, *n* (%)								
0	9 (15.0)	17 (15.7)	22 (22.0)	0.005	6 (4.4)	13 (8.1)	24 (17.3)	0.001
1–2	31 (51.7)	76 (70.4)	66 (66.0)		60 (44.4)	73 (45.3)	70 (50.4)	
3 or more	20 (33.3)	15 (13.9)	12 (12.0)		69 (51.1)	75 (46.6)	45 (32.4)	
Ave. no. of disease, mean ± SD	1.97 ± 1.40	1.45 ± 0.98	1.33 ± 1.13	0.002	2.86 ± 1.79	2.54 ± 1.58	1.96 ± 1.52	0.000
Number of medications, *n* (%)								
0	11 (18.3)	22 (20.4)	29 (29.0)	0.008	19 (14.1)	18 (11.2)	33 (23.7)	0.001
1–2	35 (58.3)	77 (71.3)	64 (64.0)		59 (43.7)	99 (61.5)	71 (51.1)	
3 or more	14 (23.3)	9 (8.3)	7 (7.0)		57 (42.2)	44 (27.3)	35 (25.2)	
Ave. no. of medication, mean ± SD	1.72 ± 1.31	1.25 ± 0.87	1.14 ± 1.04	0.003	2.36 ± 1.71	2.06 ± 1.40	1.67 ± 1.45	0.001
Activities of daily living (ADL)								
Requires no assistance	32 (53.3)	85(78.7)	92(92.0)	0.000	66 (48.9)	109 (67.7)	112 (80.6)	0.000
Some assistance needed	28 (46.7)	23(21.3)	8(8.0)		69 (51.1)	52 (32.3)	27 (19.4)	
Transportation ^(2)^, *n* (%)								
Walking only	29 (48.3)	20 (18.5)	5 (5.0)	0.000	60 (44.4)	63 (39.1)	15 (10.8)	0.000
Bus	30 (50.0)	51 (47.2)	29 (29.0)		69 (51.1)	88 (54.7)	63 (45.3)	
Subway	12 (20.0)	37 (34.3)	44 (44.0)		29 (21.5)	58 (36.0)	47 (33.8)	
Car or taxi	4 (6.7)	35 (32.4)	37 (37.0)		20 (14.8)	13 (8.1)	36 (25.9)	
Etc. ^(3)^	2 (3.3)	5 (4.6)	7 (7.0)		3 (2.2)	4 (2.5)	1 (0.7)	
Experience of social support by government ^(4)^, *n* (%)								
Yes	8 (13.3)	12 (11.1)	2 (2.0)	0.015	39 (28.9)	21 (13.0)	11 (7.9)	0.000
No	52 (86.7)	96 (88.9)	98 (98.0)		96 (71.1)	140 (87.0)	128 (92.1)	
Experience of nutrition counseling, *n* (%)								
Yes	7 (11.7)	24 (22.2)	26 (26.0)	0.095	21 (15.6)	30 (18.6)	45 (32.4)	0.001
No	53 (88.3)	84 (77.8)	74 (74.0)		114 (84.4)	131 (81.4)	94 (67.6)	

BMI = body mass index; ADL **=** activities of daily living; NQ-E = nutritional quotient for elderly; SD, standard deviation; ^(1)^ normal: ≤ 22.99 kg/m^2^, overweight: 23.00–24.99 kg/m^2^, obesity: ≥25.00 kg/m^2^; ^(2)^ multiple response; ^(3)^ Etc.: bicycle, motorcycle, welfare center vehicle, elderly electric cart, and truck; ^(4)^ social support: social care services for the elderly, home-delivered meal service, free meal service (elderly welfare facility, home support service center for the elderly, etc.); and *p*-values were determined using an independent *t*-test, one-way ANOVA test, scheffe’s test (post hoc test), or chi-square test.

**Table 4:** Living environmental factors of the subjects according to NQ-E level.

	NQ-E levels (*n* = 703)							
	Men				Women			
	Low (*n* = 60)	Medium (*n* = 108)	High (*n* = 100)	*p*-Value	Low (*n* = 135)	Medium (*n* = 161)	High (*n* = 139)	*p*-Value
Current living status, *n* (%)								
Alone	23 (38.3)	13 (12.0)	2 (2.0)	0.000	89 (65.9)	61 (37.9)	28 (20.1)	0.000
Living with spouse	31 (51.7)	88 (81.5)	91 (91.0)		23 (17.0)	61 (37.9)	91 (65.5)	
With children or children family	6 (10.0)	7 (6.5)	7 (7.0)		23 (17.0)	39 (24.2)	20 (14.4)	
Social relations, *n* (%)								
At least 3 times/week	9 (15.0)	56 (51.9)	54 (54.0)	0.000	47 (34.8)	73 (45.3)	93 (66.9)	0.000
1–2 times/week	14 (23.3)	15 (13.9)	29 (29.0)		23 (17.0)	35 (21.7)	27 (19.4)	
Less than 3 times/month	17 (28.3)	24 (22.2)	16 (16.0)		27 (20.0)	38 (23.6)	18 (12.9)	
Almost none	20 (33.3)	13 (12.0)	1 (1.0)		38 (28.1)	15 (9.3)	1 (0.7)	
Social activity ^(1)^, *n* (%)								
Resting and hobby activities	28 (46.7)	19 (17.6)	11 (11.0)	0.000	53 (39.3)	35 (21.7)	10 (7.2)	0.000
Cultural and artistic activities	5 (8.3)	6 (5.6)	12 (12.0)		16 (11.9)	19 (11.8)	23 (16.5)	
Sports activities	15 (25.0)	52 (48.1)	61 (61.0)		15 (11.1)	41 (25.5)	72 (51.8)	
Other activities	12 (20.0)	31 (28.7)	16 (16.0)		51 (37.8)	66 (41.0)	34 (24.5)	
Frequency of social activity, *n* (%)								
Rarely	34 (56.7)	26 (24.1)	5 (5.0)	0.000	80 (59.3)	54 (33.5)	11 (7.9)	0.000
Regular	18 (30.0)	33 (30.6)	44 (44.0)		22 (16.3)	53 (32.9)	45 (32.4)	
Trying to be active	8 (13.3)	49 (45.4)	51 (51.0)		33 (24.4)	54 (33.5)	83 (59.7)	
Food security, *n* (%)								
Food secure	8 (13.3)	51 (47.2)	60 (60.0)	0.000	29 (21.5)	58 (36.0)	93 (66.9)	0.000
Mildly food insecure	38 (63.3)	48 (44.4)	38 (38.0)		76 (56.3)	83 (51.6)	41 (29.5)	
Moderately/severely food insecure	14 (23.3)	9 (8.3)	2 (2.0)		30 (22.2)	20 (12.4)	5 (3.6)	
Ability to cook, *n* (%)								
Yes	38 (63.3)	63 (58.3)	64 (64.0)	0.668	115 (85.2)	147 (91.3)	133 (95.7)	0.010
No	22 (36.7)	45 (41.7)	36 (36.0)		20 (14.8)	14 (8.7)	6 (4.3)	
Nutrition knowledge ^(2)^, *n* (%)								
Low	29 (48.3)	17 (15.7)	15 (15.0)	0.000	58 (43.0)	27 (16.8)	3 (2.2)	0.000
Medium	25 (41.7)	69 (63.9)	41 (41.0)		63 (46.7)	97 (60.2)	85 (61.2)	
High	6 (10.0)	22 (20.4)	44 (44.0)		14 (10.4)	37 (23.0)	51 (36.7)	
Average nutrition knowledge, mean ± SD	3.57 ± 3.01	5.56 ± 2.50	6.34 ± 2.70	0.000	3.99 ± 2.76	5.85 ± 2.71	6.78 ± 1.75	0.000

NQ-E, nutritional quotient for elderly; SD, standard deviation; ^(1)^ resting and hobby activities: listening to music, reading/newspaper/magazine, napping, sauna, gardening, fishing, baduk/Korean chess (chang gi), cooking, etc.; cultural and artistic activities: movies, musical instruments, singing classes, arts, dancing, exhibition/museums, concerts, traditional art, etc.; sport activities: walking, badminton, ping-pong, swimming, running, billiards, etc.; other activities: religion, reunions, visiting relatives, speaking on the telephone, welfare facility, volunteer, etc.; ^(2)^ low: 0–3 score, medium: 4–7 score, and high: 8–10 score; and *p*-values were determined using an independent *t*-test, one-way ANOVA test, scheffe’s test (post hoc test), or chi-square test.

**Table 5:** Multinomial logistic regressions of an association between living environmental factors and NQ-E levels in elderly men.

	Men (*n* = 268)					
	Medium-NQ-E			High-NQ-E		
	Crude OR (95% CI)	Model 1 ^(1)^ OR (95% CI)	Model 2 ^(2)^ OR (95% CI)	Crude OR (95% CI)	Model 1 OR (95% CI)	Model 2 OR (95% CI)
Current living status, *n* (%)						
Alone	1.00	1.00	1.00	1.00	1.00	1.00
Living with spouse	5.02 *** (2.27–11.10)	3.10 * (1.21–7.93)	2.74 (0.97–7.75)	33.75 *** (7.52–151.48)	11.75 ** (2.24–61.67)	8.99 * (1.35–59.56)
With children or children family	2.06 (0.57–7.46)	0.68 (0.14–3.31)	0.40 (0.07–2.22)	13.41 ** (2.19–81.99)	3.02 (0.36–24.90)	1.41 (0.12–15.74)
Social relations, *n* (%)						
At least 3 times/week	9.57 *** (3.55–25.80)	2.60 (0.71–9.50)	1.75 (0.41–7.43)	120.00 *** (14.27–1008.50)	15.34 * (1.31–179.57)	8.26 (0.56–121.56)
1–2 times/week	1.64 (0.60–4.52)	0.60 (0.16–2.18)	0.35 (0.08–1.57)	41.42 ** (5.03–340.76)	8.69 (0.75–99.91)	4.42 (0.29–65.60)
Less than 3 times/month	2.17 (0.85–5.53)	1.32 (0.45–3.83)	1.54 (0.46–5.14)	18.82 ** (2.25–157.01)	7.19 (0.63–82.00)	8.78 (0.60–127.48)
Almost none	1.00	1.00	1.00	1.00	1.00	1.00
Social activity ^(3)^, *n* (%)						
Resting and hobby activities	1.00	1.00	1.00	1.00	1.00	1.00
Cultural and artistic activities	1.76 (0.47–6.63)	0.85 (0.19–3.74)	0.88 (0.16–4.83)	6.10 ** (1.74–21.42)	1.69 (0.36–7.78)	2.56 (0.43–15.16)
Sports activities	5.10 *** (2.25–11.57)	2.89 * (1.10–7.53)	3.41 * (1.18–9.80)	10.35 *** (4.21–25.39)	2.41 (0.74–7.78)	2.86 (0.75–10.79)
Other activities	3.80 ** (1.57–9.22)	1.47 (0.48–4.42)	1.16 (0.34–3.94)	3.39 * (1.22–9.44)	1.31 (0.34–4.93)	0.94 (0.22–3.98)
Frequency of social activity, *n* (%)						
Rarely	1.00	1.00	1.00	1.00	1.00	1.00
Normal	2.39 * (1.11–5.17)	1.23 (0.49–3.04)	0.83 (0.30–2.27)	16.62 *** (5.60–49.30)	5.77 ** (1.61–20.73)	2.56 (0.65–10.12)
Trying to be active	8.01 *** (3.24–19.80)	2.85 (0.96–8.43)	2.26 (0.70–7.29)	43.35 *** (13.07–143.73)	9.04 ** (2.20–37.08)	3.93 (0.86–17.85)
Food security, *n* (%)						
Food secure	9.91 *** (3.23–30.42)	4.81 *(1.31–17.68)	3.96 (0.87–17.95)	52.50 *** (10.03–274.76)	14.61 ** (2.08–102.16)	6.57 (0.73–58.98)
Mildly food insecure	1.96 (0.76–5.02)	1.29 (0.43–3.82)	0.71 (0.18–2.73)	7.00 * (1.48–32.92)	3.22 (0.51–20.06)	1.02 (0.12–8.29)
Moderately/severely food insecure	1.00	1.00	1.00	1.00	1.00	1.00
Nutrition knowledge ^(4)^, *n* (%)						
Low	1.00	1.00	1.00	1.00	1.00	1.00
Medium	4.70 *** (2.21–10.00)	2.37 (0.95–5.90)	2.32 (0.82–6.51)	3.17 ** (1.42–7.03)	0.74 (0.24–2.26)	0.49 (0.13–1.82)
High	6.25 ** (2.11–18.47)	1.95 (0.49–7.70)	1.71 (0.35–8.31)	14.17 *** (4.92–40.77)	1.16 (0.27–4.92)	0.76 (0.13–4.21)

95% CI, 95% confidence interval; NQ-E, nutritional quotient for elderly; OR, odds ratio.; ^(1)^ model 1: adjusted for age, education, and monthly income; ^(2)^ model 2: adjusted for age, education, monthly income, BMI, number of diseases, activities of daily living, experience of social support by government, food security, and nutrition knowledge; ^(3)^ resting and hobby activities: listening to music, reading/newspaper/magazine, napping, sauna, gardening, fishing, baduk/Korean chess (changgi)/yut, senior classes, cooking, etc.; cultural and artistic activities: movies, musical instruments, singing classes, art, dancing, exhibition/museums, concerts, traditional art, photography, etc.; sport activities: walking, badminton, ping-pong, swimming, running, billiards, etc.; other activities: religion, reunions, visiting relatives, speaking on the telephone, welfare facility, volunteer, etc.; ^(4)^ low: 0–3 score, medium: 4–7 score, and high: 8–10 score; and *p*-values were determined using a multinomial logistic regressions analysis (* *p* < 0.05, ** *p* < 0.01, and *** *p* < 0.001).

**Table 6:** Multinomial logistic regressions of an association between living environmental factors and NQ-E levels in elderly women.

	Men (*n* = 268)					
	Medium-NQ-E			High-NQ-E		
	Crude OR (95% CI)	Model 1 ^(1)^ OR (95% CI)	Model 2 ^(2)^ OR (95% CI)	Crude OR (95% CI)	Model 1 OR (95% CI)	Model 2 OR (95% CI)
Current living status, *n* (%)						
Alone	1.00	1.00	1.00	1.00	1.00	1.00
Living with spouse	3.87 *** (2.16–6.91)	3.13 *** (1.65–5.92)	3.11 ** (1.52–6.35)	12.57 *** (6.73–23.47)	5.44 *** (2.61–11.33)	5.62 *** (2.36–13.38)
With children or children family	2.47 ** (1.34–4.55)	2.08 * (1.08–3.97)	1.95 (0.96–3.96)	2.76 ** (1.32–5.76)	1.70 (0.73–3.93)	1.88 (0.72–4.88)
Social relations, *n* (%)						
At least 3 times/week	3.56 ** (1.64–7.73)	2.86 ** (1.35–6.08)	2.35 * (1.02–5.40)	25.33 ** (3.18–201.41)	24.94 ** (3.10–200.17)	16.04 * (1.72–149.18)
1–2 times/week	3.85 ** (1.73–8.54)	2.22 (0.92–5.31)	2.07 (0.81–5.31)	44.60 *** (5.67–350.69)	10.25 * (1.19–88.33)	9.80 (0.99–96.57)
Less than 3 times/month	3.93 *** (1.95–7.93)	2.88 * (1.28–6.47)	2.70 * (1.12–6.51)	75.19 *** (10.01–564.74)	11.03 * (1.28–94.59)	9.42 (0.94–93.74)
Almost none	1.00	1.00	1.00	1.00	1.00	1.00
Social activity ^(3)^, *n* (%)						
Resting and hobby activities	1.00	1.00	1.00	1.00	1.00	1.00
Cultural and artistic activities	1.79 (0.81–3.96)	1.45 (0.62–3.36)	1.56 (0.61–3.98)	7.61 *** (3.00–19.29)	3.59 * (1.21–10.65)	5.11 * (1.46–17.82)
Sports activities	4.13 *** (1.99–8.58)	3.40 ** (1.56–7.43)	2.98 * (1.28–6.91)	25.44 *** (10.60–61.04)	13.91 *** (5.06–38.17)	12.07 *** (3.91–37.20)
Other activities	1.96 * (1.11–3.43)	1.47 (0.79–2.74)	1.46 (0.75–2.87)	3.53 ** (1.58–7.88)	2.34 (0.92–5.91)	2.32 (0.81–6.58)
Frequency of social activity, *n* (%)						
Rarely	1.00	1.00	1.00	1.00	1.00	1.00
Normal	3.56 ***(1.94–6.53)	3.07 **(1.62–5.83)	2.64 **(1.30–5.38)	14.87 ***(6.61–33.46)	7.75 ***(3.14–19.11)	5.75 **(2.09–15.76)
Trying to be active	2.42 **(1.39–4.21)	1.95 *(1.07–3.53)	1.41(0.72–2.75)	18.29 ***(8.65–38.65)	9.45 ***(4.11–21.71)	5.28 **(2.05–13.58)
Food security, *n* (%)						
Food secure	3.00 **(1.46–6.16)	2.81 **(1.29–6.13)	1.79 (0.73–4.41)	19.241 ***(6.83–54.137)	10.79 ***(3.44–33.86)	5.07 *(1.35–18.97)
Mildly food insecure	1.63 (0.85–3.12)	1.52 (0.76–3.04)	1.40 (0.64–3.06)	3.23 *(1.16–8.97)	2.28 (0.74–7.01)	1.98 (0.54–7.17)
Moderately/severely food insecure	1.00	1.00	1.00	1.00	1.00	1.00
Nutrition knowledge ^(4)^, *n* (%)						
Low	1.00	1.00	1.00	1.00	1.00	1.00
Medium	3.30 *** (1.89–5.76)	2.87 ** (1.58–5.20)	2.61 ** (1.36–5.00)	26.08 *** (7.81–87.06)	19.86 *** (5.17–76.15)	17.71 *** (4.14–75.763)
High	5.67 *** (2.63–12.21)	3.52 ** (1.52–8.17)	3.50 ** (1.35–9.03)	70.42 *** (19.14–259.06)	23.61 *** (5.46–102.12)	20.49 *** (4.12–101.73)

95% CI, 95% confidence interval; NQ-E, nutritional quotient for elderly; OR, odds ratio; ^(1)^ model 1: adjusted for age, education, and monthly income; ^(2)^ model 2: adjusted for age, education, monthly income, BMI, number of diseases, activities of daily living, experience of social support by government, food security, and nutrition knowledge; ^(3)^ resting and hobby activities: listening to music, reading/newspaper/magazine, napping, sauna, gardening, fishing, baduk/Korean chess (changgi)/yut, senior classes, cooking, etc.; cultural and artistic activities: movies, musical instruments, singing classes, art, dancing, exhibition/museums, concerts, traditional art, photography, etc.; sport activities: walking, badminton, ping-pong, swimming, running, billiards, etc.; other activities: religion, reunions, visiting relatives, speaking on the telephone, welfare facility, volunteer, etc.; ^(4)^ low: 0–3 score, medium: 4–7 score, and high: 8–10 score; and *p*-values were determined using a multinomial logistic regressions analysis (* *p* < 0.05, ** *p* < 0.01, and *** *p* < 0.001).
